# Experimental and theoretical studies of linear and non-linear optical properties of novel fused-triazine derivatives for advanced technological applications

**DOI:** 10.1038/s41598-022-22311-z

**Published:** 2022-11-19

**Authors:** Hamdan A. S. Al-Shamiri, Mahmoud E. M. Sakr, Samir A. Abdel-Latif, Nabel A. Negm, Maram T. H. Abou Kana, Samy A. El-Daly, Ahmed H. M. Elwahy

**Affiliations:** 1 Physics Department, Faculty of Science, University of Bisha, P.O. Box 551, Bisha, 61922 Saudi Arabia; 2 Physics Department, Faculty of Applied Science, Taiz University, P.O. Box 4007, Taiz, Yemen; 3 Laser Sciences and Interactions Department, National Institute of Laser-Enhanced Sciences (NILES), Cairo University, Giza, Egypt; 4 Department of Chemistry, Faculty of Science, Helwan University, Cairo, 11795 Egypt; 5 Egyptian Petroleum Research Institute (EPRI), Nasr City, 11727 Cairo Egypt; 6 Chemistry Department, Faculty of Science, Tanta University, Tanta, Egypt; 7 Chemistry Department, Faculty of Science, Cairo University, Giza, Egypt

**Keywords:** Chemistry, Optics and photonics

## Abstract

Controlling photophysical properties is critical for the continued development of electroluminescent devices and luminescent materials. The preparation and study of novel molecules suitable as luminescent for the development of optoelectrical devices have recently received a lot of attention. Even though the as-triazine unit is a good building block for organic active substances, it is rarely used in this context. We created here novel bis-triazine derivative dyes in the far UV–Vis range by alkylation of triazine-thione derivatives with appropriate dibromo compounds. At the B3LYP/6-311**G(d,p) basis set, their optimal molecular structures were obtained. DFT technique confirmed that the new triazine derivatives are in noncoplanar with one of the two phenyl rings and the triazine plane rotating out by 102.09. Also, depending on the energy gap difference between HOMO and LUMO, some important parameters including chemical potential (π), electronegativity (χ), and chemical hardness (η) were calculated. The compounds may be readily polarized and have significant NLO characteristics, as seen by the tiny HOMO–LUMO energy gap. The calculated values for the polarizability (α) of the two new triazine derivatives have the range 6.09–10.75 × 10^–24^ (esu). The emission peaks seemed to move to the long-wavelength (redshift), with a rise in the fluorescence band, suggesting that the singlet excited state is more polar than the ground state. The influence of solvent polarity and the intermolecular charge transfer (ICT) processes are reflected in the photophysical properties of new fused triazine derivatives. These properties such as extinction coefficient, absorption and emission cross-sections, fluorescence quantum yield, fluorescence lifetime, oscillator strength, the dipole moment, radiative decay rate constant, the energy yield of fluorescence, and the attenuation length were assessed and discussed.

## Introduction

The development of novel organic luminophores has been extensively investigated due to their wide-ranging applications in domains such as bioimaging, optical storage, and optoelectronics^1–4^. Additional uses of these photochemical organic materials include organic light-emitting diodes (OLEDs)^5^, phosphorescent probes^6^, and dye-enhanced solar cells^7^. The time of conjugation is typically controlled in operation to efficiently maximize the luminescence characteristics of organic molecules throughout a particular range of optical wavelength regions. The presence of both electron substituents that donate (D) and accept (A) in a single molecule also exposes interesting spectral and optical characteristics owing to intramolecular charge transfer (ICT). The 1,2,4-triazine unit has been used as a building block for organic active materials, particularly organic light-emitting diodes^8,9^. In this regard, Xiang et al.^10^ demonstrated the potential of as-triazines as luminescent materials. Furthermore, Maggiore et al. recently reported the use of some fused 1,2,4-triazine systems as chromophores that exhibit both thermally-activated delayed fluorescence (TADF) and crystallization-induced phosphorescence^11^. Spectroscopic methods and the density functional theory (DFT) have recently been used to identify the structures of a range of organic molecules^12,13^. In previous work^14^, we synthesized various bis-triazines with various spacers as chromophores. DFT and the time-dependent density functional theory (TD-DFT) studies confirmed that the spacer had a significant effect on their highest occupied molecular orbital (HOMO) and lowest unoccupied molecular orbital (LUMO) energy gaps and other simulated characterizations. Because of the lack of conjugation, these compounds did not exhibit the optical behavior required for advanced technological applications. The current study, which is a continuation of our interest in this field, focus on the synthesis of novel bis-fused triazines with an extended π-conjugated system. Spectroscopic methods were used to confirm their chemical structures. The ground state features such as geometrical parameters, optimization structure, reactivity parameters, and 3D plots of the molecule electrostatic potential maps will be studied using theoretical calculations using (DFT) at the basis set B3LYP/6-311 G(d,p) (MEP). The polarizable continuous solvation model will be used to investigate the derivation of electronic spectra and the composition of the frontier molecular orbitals using TD-DFT (PCM). The optical characteristics and photophysical parameters of various solvent polarities were also experimentally and theoretically described.

## Experimental

### General

1,4-Dibromobutane and 1,4-bis(bromomethyl)benzene were purchased from Sigma-Aldrich and used without purification. Melting points were assessed in open glass capillaries using a Gallenkamp apparatus. The infrared spectra were recorded using a PyeUnicam SP3-300 and Shimadzu FTIR 8101 PC infrared spectrophotometer. ^1^H NMR spectra were obtained by using a Varian Mercury VX 300 NMR spectrometer (TMS used as an internal standard and DMSO-d_6_ as a solvent). Mass spectra were determined using a GCMS-QP1000 EX spectrometer at 70 eV. Elemental analyses were carried out at the Microanalytical Center of Cairo University, Giza, Egypt.

### Synthesis of bis-triazine compounds 3 and 5

The appropriate dibromo compounds **2** and **4** were added to a solution of phenanthro[9,10-*e*][1,2,4]triazine-3(4*H*)-thione **1** (1 mmol) and KOH (1 mmol) in absolute ethanol (10 ml). The reaction mixture was heated at reflux for 1 h, then allowed to cool to room temperature. The formed crude solid was filtered off, dried in a vacuum oven, and recrystallized using dimethylformamide (DMF) giving yellow crystals of **3** and **5**, respectively^14^.

#### 1,4-Bis(phenanthro[9,10-e][1,2,4]triazin-3-ylthio)butane (3)

With the use of the general procedure, compound **1** and 1,4-Dibromobutane **2** gave crude **3** which crystallized from DMF as yellow crystals (77%), mp 246–248 °C; IR: υ max 3063 (CH), 1602, 1485 (C = C) cm^−1^, ^1^H NMR (DMSO) δ 2.16 (br, 4H, SCH_2_CH_2_), 3.50 (br, 4H, SCH_2_CH_2_), 7.49–7.83 (m, 8H, ArHs), 8.46–9.06 (m, 8H, ArHs) ppm. MS: m/z 580 (M^+^); Anal. for C_34_H_24_N_6_S_2_, Calcd. C, 70.32; H, 4.17; N, 14.47. Found: C, 70.70; H, 4.30; N, 14.70.

#### 1,4-Bis((phenanthro[9,10-e][1,2,4]triazin-3-ylthio)methyl)benzene (5)

With the use of the general procedure, compound 1 and the potassium salt of **4** gave crude **5**, which crystallized from DMF as yellow crystals (81%), mp 278–280 °C; IR: υ max 3069 (CH), 1603, 1478 (C=C) cm^−1^, ^1^H NMR (DMSO) δ 4.67 (s, 4H, SCH_2_), 7.45–7.60 (m, 8H, ArHs), 7.65 (s, 4H, ArHs), 8.20–8.80 (m, 8H, ArHs) ppm. MS: m/z 628 (M^+^); Anal. for C_38_H_24_N_6_S_2_, Calcd. C, 72.59; H, 3.85; N, 13.37. Found: C, 72.70; H, 4.10; N, 13.10.

### Spectral measurements

Solutions of newly prepared dyes with concentrations ranging from 4 × 10^–6^ to 2 × 10^–5^ M in *N,N*-dimethylformamide (DMF) were contained in quartz cells of optical path sealed carefully during the measurements. The optimum concentration was 1 × 10^–5^ M in *N*,*N*-dimethylformamide (DMF). The optimum concentration of prepared probes was dissolved into different solvents such as cyclohexane, tetrahydrofuran (THF), chloroform, dimethylsulfoxide (DMSO), ethanol, and methanol. These fluorophores' absorption and fluorescence characteristics in various solvents were investigated.

A Camspec M501 UV/Vis spectrophotometer and a PF-6300 spectrofluorometer were used to measure the absorption and excitation spectra, respectively. The absorption and emission spectra of compounds 3 and 5 in DMF were used to determine the optimal concentration.

### Photophysical parameters calculations

For deep explanations of the spectroscopic behavior of new dyes, some photo-physical parameters have been assessed. These parameters included the dipole moment transition (µ_12_)^15^, the decay of excited electrons either radiative and/or non-radiative^16^, cross-sections of absorption and emission^17^, quantum yield^18,19^. Also, the molar absorption coefficient function is presented by oscillator strength^20^. The attenuation length Λ (λ)^21^ and excited state (τ_f_)^22–25^ have been estimated.

### Computational details

Energy minimization analyses were performed using the Gaussian-09 W software program^26^ because of the lack of single-crystal X-ray structure analysis to get the molecular conformation of the produced compounds. The ground state geometrical structures the compounds **3** and **5** were optimized using the density functional theory with Becke's three-parameter exchange functional method^27^, the Lee–Yang–Parr correlation functional (B3LYP), and, the split-valence double zeta basis set with two polarized basis functions (d and p), (DFT/B3LYP) at the 6-311G(d,p) with the B3LYP exchange–correlation approach^28^. For C, H, N, and O atoms, the basis set 6-311G(d,p) was used^29,30^.

Every bond length, bond angle, and dihedral angle could relax free of restrictions thanks to geometry optimizations, and the geometry of the investigated systems was completely optimized in the gas phase.

The DFT theory may be used to examine a variety of characteristics, including optimization energy, geometrical parameters, 3D plots of molecular electrostatic potential maps (MEP), and reactivity parameters. Gauss-View 5 software^31^, Avogadro^32^, and Chemcraft^33^ programs have been used to extract the calculated results and visualize the optimized forms, the frontier molecular orbitals, and 3D plots of the molecular electrostatic potential (MEP) maps. The quantum chemical parameters of the compounds are gained from these equations^34,35^; E_g_ = E_LUMO_ − E_HOMO_, χ = − E_HOMO_ + E_LUMO_/2, η = E_LUMO_ − E_HOMO_/2, σ = 1/η, π = − χ, S = 1/2η, ω = π^2^/2η and ΔN_max_. = − π/η. To explain their optical characteristics, spin density difference map calculations were also conducted.

To explore distinct second-order interactions between the filled orbital of one subsystem and the unoccupied orbital of another, natural bond orbital (NBO) computations were conducted^36^ utilizing the NBO code included in Gaussian 09's NBO code. The mean polarizability (<α>), the anisotropy of the polarizability (Δα), the mean first-order hyperpolarizability (<β, >) and the total static dipole-moment (μ) via the x, y, z components were analyzed^37,38^. To elucidate the origin of electronic spectra, TD-DFT computations were performed at the same level of theory (B3LYP/6-31G(d,p)) using the polarizable continuous solvation technique PCM, PCM-TD-DFT. Gauss View 5 software^34^ was used to prepare the figures showing the molecular orbitals (MOs).

## Results and discussion

### Synthesis

**Figure 1:**
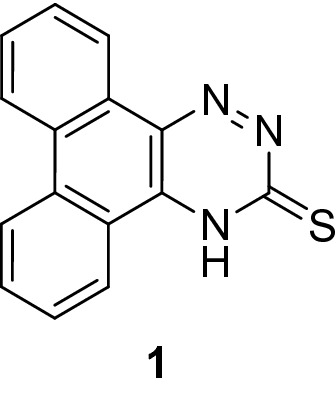
Structure of compound **1**.

As previously disclosed^39^, the starting compound, phenanthro[9,10-*e*][1,2,4]triazine-3(4*H*)-thione **1** was produced in excellent yield by the reaction of phenanthraquinon with thiosemicarbazide in basic medium (Fig. 1).

**Scheme 1:**
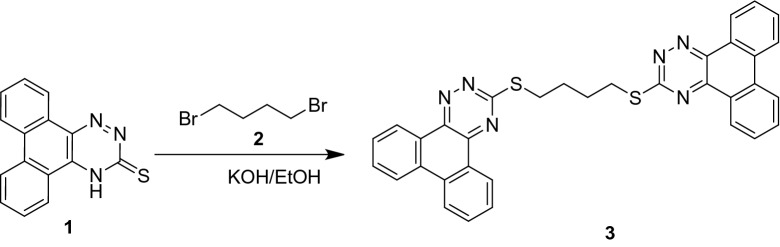
Synthesis of compound 3.

**Scheme 2:**
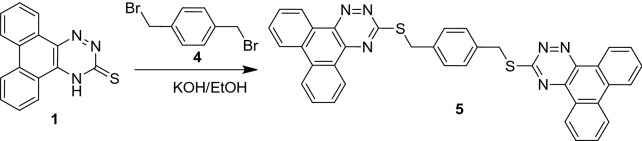
Synthesis of compounds **5**.

As shown in Schemes 1 and 2, the synthetic usefulness of **1** as a building block for new bis(phenanthro[9,10-*e*][1,2,4]triazine)s **3** and **5** was explored. In ethanol containing KOH at reflux, treatment of **1** with 1,4-dibromobutane **2** yielded 1,4-bis(phenanthro[9,10-*e*][1,2,4]triazin-3-ylthio)butane (3) in 77% yield (Scheme 1).

Using a similar method, 1,4-bis((phenanthro[9,10-*e*][1,2,4]triazin-3-ylthio)methyl)benzene **5** was obtained in 81% yield by reacting **1** with 2,6-bis(bromomethyl)benzene 4 in ethanol containing KOH at refluxing temperature (Scheme 2).

Elemental studies and spectrum data were used to describe all the isolated compounds, and they all agreed with the hypothesized structures. IR, 1H-NMR, and mass spectra were used to corroborate the structures of bis(phenanthro[9,10-*e*][1,2,4]triazines) **3** and **5**. In the IR spectrum of **3** and **5**, the absence of an absorption band corresponding to the parent dihydrophenanthro[9,10-*e*][1,2,4]triazine-3(4*H*)-thione **1**'s NH or C=S stretching frequencies certified the production of bis(phenanthro[9,10-*e*][1,2,4]triazine) derivatives. Furthermore, the 1H NMR spectra of compound **3** revealed two wide signals at 2.16 and 3.50, each integrated for four protons, which were attributed to the butylene spacer's methyl protons. Compound **5**'s 1H NMR spectra revealed a singlet signal at 4.67, which is typical of OCH_2_ protons. The chemical shifts and integral values of all other protons were as predicted. Compounds **3** and **5** had strong molecular ion peaks at m/z 586 and 628, respectively, which matched their molecular formulas.

**Scheme 3:**
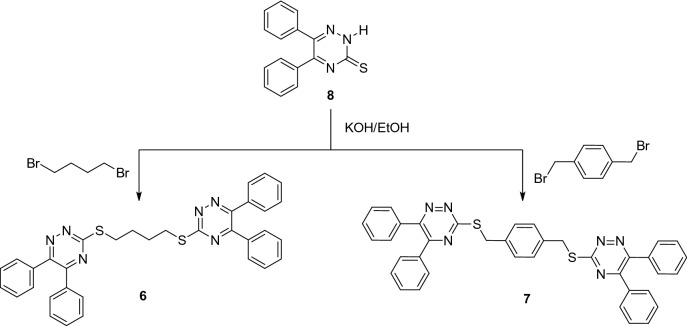
Synthesis of compounds **6** and **7**.

It is worth noting that we have recently synthesized bis-triazines **6** and **7** from 5,6-diphenyl-1,2,4-triazine-3(2*H*)-thione **8**. Their absorption and excited-emission spectra, as well as molecular structure optimization using the B3LYP/6–31G(d) level of theory, were investigated (Scheme 3)^14^.

### DFT calculation

**Figure 2:**
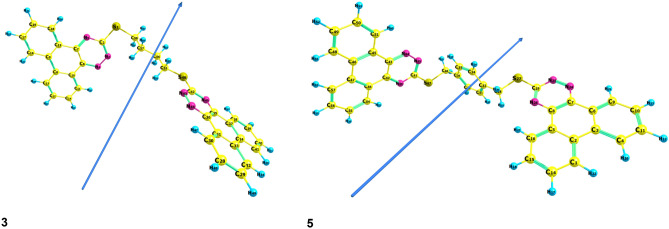
Optimized geometry, numbering system, and vector of dipole moment of **3** and **5** using B3LYP/ 6-311G**.

The DFT technique was used to determine the electronic ground state structures of compounds **3** and **5**. Figure 2 shows the outcomes of the optimizations performed at the B3LYP /6-311G** level of theory. To avoid steric hindrance, compounds **3** and **5** are noncoplanar, with one of the two phenyl rings and the triazine plane rotating out by 102.09. The single bond character is shown by the lengths of the C–C bonds linking the two phenyl rings and the triazine ring, which are 1.418 and 1.417, respectively. Figure 2 shows that this is true for both HOMO and LUMO molecular orbitals.

**Figure 3:**
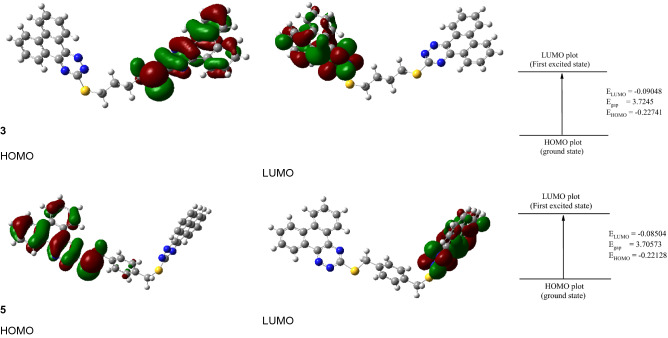
HOMO and LUMO charge density maps of the studied **3** and **5** using B3LYP/6-311G** level.

The HOMO molecular orbitals are concentrated in one portion of the molecule (over the triazine phenyl rings), while the LUMO is delocalized in the other. As a result, there is little interaction between the numerous sub-systems in each molecule, which is reflected in the UV spectra. As illustrated in Fig. 3, both HOMO and LUOM molecular orbitals are focused on certain sub-systems of the two molecules. Table 1 lists the geometrical characteristics of the **3** and **5** compounds in gas form. Figure 2 depicts the labeling scheme. The variation in bond lengths or angles of the triazine moiety in compounds **3** and **5** is seen in Table 1.

**Table 1:** Selected geometric bond lengths, bond angles, and, dihedral angles of the optimized **3** and **5** compounds using B3LYP/6-311G**.

Compound	Bond lengths (Å)		Bond angles		Dihedral angles	
**3**	C6-C7	1.4176	N3-C2-N5	126.140	S1-C2-N5-C7	179.104
	N4-C6	1.3444	C2-N2-N3	30.623	S1-C2-N3-N4	− 178.893
	N5-C7	1.3382	N3-N4-C6	119.970	C2-N3-N4-C6	− 0.206
	C2-N3	1.3509	N4-C6-C7	120.217	N3-N4-C6-C7	− 0.223
	C2-N5	1.3323	C6-C7-N5	119.688	N4-C6-C7-N5	0.410
	C2-S1	1.7715	N3-C2-S1	118.954	C6-C7-N5-C2	− 0.143
	S1-C58	1.8389	C2-S1-C58	39.573	S1-C56-C57-C58	− 178.012
	C55-S46	1.8414	C42-S46-C55	102.091	C55-S46-C42-N43	− 0.840
	C42-S46	1.7687	S46-C42-N43	118.573	S46-C42-N43-N44	179.894
	C42-N43	1.3521	N43-C42-N45	126.223	C42-N43-N44-C36	− 0.032
	C42-N45	1.3327	C42-N45-C35	115.979	N43-N44-C36-C35	0.116
	C35-N45	1.3380	N45-C35-C36	119.700	N44-C36-C35-N45	− 0.066
	C36-N44	1.3446	C35-C36-N44	120.240	C36-C35-N45-C42	− 0.066
**5**	C19-N25	1.3594	N24-N25-C19	117.858	N24-N25-C19-N26	− 0.067
	C19-N26	1.3268	C7-N24-N25	120.041	C7-N24-N25-C19	0.017
	N24-N25	1.3109	C7-C8-N26	119.669	C7-C8-N26-C19	0.061
	C7-N25	1.3517	C7-C8-C5	120.291	C7-C8-C5-C2	− 0.168
	C19-S27	1.7699	C28-C32-C31	120.705	C28-C32-C31-C33	179.867
	S27-C28	1.8517	C32-C31-C33	120.692	C32-C31-C33-C36	0.092
	S67-C68	1.8561	C41-N65-N64	118.010	C41-N65-N64-C43	− 0.097
	S67-C41	1.7710	N65-N64-C43	119.972	N65-N64-C43-C42	0.080
	C41-N65	1.3503	N64-C43-C42	120.197	N64-C43-C42-N66	0.019
	C41-N66	1.3324	C43-C42-N66	119.666	C43-C42-N66-C41	− 0.092

Figure 3 depicts the HOMO and LUMO, orbitals, and energy gap between HOMO and LUMO (Eg) for three and five compounds in gas at the B3LYB/6-311G** level of theory.

These molecular orbitals are primarily concentrated over certain parts of the molecule (triazine and phenyl groups), rather than being stretched across the full molecule. Table 2 also includes the computed HOMO and LUMO energy values, as well as the energy gap between HOMO and LUMO (Eg) of the investigated compounds. The estimated Eg of the investigated compounds rises in sequence **5** < **3**, indicating that compound **5** has the highest reactivity and compound **3** has the lowest.

**Table 2:** Total energy, the energy of HOMO and LUMO, energy gap, ionization energy (I, eV), electron affinity (A, eV), absolute electronegativities, (χ, eV), absolute hardness (η, eV), global softness (S, eV^−1^) chemical potential (π*,* eV^−1^of **3** and **5** using B3LYP/6-311G**.

Parameter	**3**	**5**
E_T_, a.u	− 2434.98	− 2587.40
E_HOMO_, a.u	− 0.2274	− 0.2213
E_LUMO_, a.u	− 0.0904	− 0.0850
E_g_, eV	3.7280	3.7089
I, eV	6.1879	6.0219
A, eV	2.4599	2.3130
χ, eV	4.3239	4.1675
η, eV	1.8640	1.8545
S, eV	0.2682	0.2696
π, eV	− 4.3239	− 4.1675

The I.P., or ability to lose electrons, is equivalent to -EHOMO, therefore the I.P. values are in this order: **5** < **3** ELUMO, on the other hand, is connected to the compound's electron affinity (E.A) by the equation E.A. = ELUMO. The order of E.A. values in Table 2 is **5** < **3**; that is, compound 3 has the highest inclination to receive electrons.

Chemical reactivity, kinetic stability, and biological activity of molecular systems are all measured using the energy gap between frontier molecular orbitals (HOMO and LUMO).

The difference [ELUMO-EHOMO] determines the energy gap (Eg). The estimated Eg of the investigated triazine compounds rises in the sequence **5** < **3**, indicating that **5** has greater reactivity. The lowering of Eg value of **5** compared to that of **3** indicates less stability, a significant effect of intramolecular charge transfer (ICT), consequently, the absorption spectra are red-shifted. Another important calculated parameter, using E_LUMO_ and E_HOMO_ values, is the chemical potential (π), electronegativity, and chemical hardness (η). These parameters are calculated as follows μ = E_HOMO_ + E_LUMO_/2^40^, χ = − E_HOMO_ + E_LUMO_/2^38^ η = E_LUMO_ − E_HOMO_/2^38^ and chemical softness (S = 1/ η). Also, compound 3 has a high χ value than compound **5** (Table 2), thus molecule **3** is the one that can attract the electrons from other compounds. On another side, compound **3** has a high η value in comparison with the other one **5**, this indicates that compound **3** is very difficult to liberate the electrons, while the other 1,2,4-triazine molecule (**5**) are good candidates to provide electrons to another acceptor molecule (see Table 2). When a molecule has a large dipole moment, the distribution of electronic charge is asymmetric, and it might be more reactive and sensitive to changes in its electronic molecular structure and electronic properties when exposed to an external electric field. The dipole moment (μ) of compound **5** is greater than that of compound **3**, as seen in Table 2.

As a result, this molecule has a higher reactivity. The lower chemical potential value, μ, of compound **3** when compared to the other **5** (see Table 2) suggests that compound **3** has a lesser tendency for electrons to drain than the other **5**^41^.

As a result, molecule **3** is thought to be the hardest, more stable, and least reactive of the two^42^.

### Nonlinear optical properties (NLO)

The distribution of the atomic charges in the molecular compounds is also respected in the determination of the extent and direction of its moment. The mean polarizability, the anisotropy of the polarizability, the dipole moment, and the first order hyperpolarizability for the studied **3** and **5** compounds, as well as urea^43^, were calculated using a similar level and the found results are tabulated in Table 3. The table also comprises the experimental estimates of urea. The calculated dipole moment value of compounds **3** and **5** are 2.76 and 6.96 D, respectively. Compound **5** has a higher dipole moment value than the other one **3** and both have higher values than that of urea. The polarizabilities and first order hyperpolarizabilities are described in atomic units (au); the calculated values have been adapted into electrostatic units (esu) using adaptation factors of 0.1482×10^−24^ esu for α and 8.6393×10^−33^ esu for β. Urea is a standard pattern used in NLO studies. In this study, urea was selected as a reference as there were no experimental standards of NLO properties of the considered compounds. The magnitude of β is one of the main aspects of an NLO system. The calculated values of the polarizability of compounds **3** and **5** have the range 6.09-10.75x10^−24^ (esu). The estimated value of compound **5** is the lowest, whereas compound **3** is the highest.

**Table 3:** Calculated total static dipole moment (μ), the mean polarizability <α> , anisotropy of the polarizability Δα and the first-order hyperpolarizability <β> configuration for the studied **3** and **5** compounds using B3LYP/6-311G**.

Property	Urea	**3**	**5**
µ, D	1.3197	2.7574	6.9628
αxx, a.u	–	− 171.116	− 221.9209
αyy	–	− 240.1205	− 259.9456
αzz	–	− 246.7902	− 265.554
αxy	–	8.1839	− 5.2275
αxz	–	7.0232	10.1345
αyz	–	0.3379	3.11
<α>, esu	–	− 3.25065 × 10^–23^	− 3.69226 × 10^–23^
Δα, esu	–	10.7548 × 10^–24^	6.09351 × 10^–24^
βxxx	–	− 122.1188	− 83.2774
βxxy	–	289.3349	445.748
βxyy	–	14.1789	65.3903
βyyy	–	12.3975	43.0987
βxxz	–	11.6289	− 61.3536
βxyz	–	− 77.4661	20.805
βyyz	–	− 5.2596	− 46.3942
βxzz	–	6.3025	88.0173
βyzz	–	24	39.3975
βzzz	–	− 4.196	10.0287
<β>, esu	0.1947 × 10^–30^	2.9450 × 10^–30^	4.6805 × 10^–30^

Compound **5** is 24 times higher than urea, whereas compound **3** is 15 times higher than the reference material, according to the study of the analysis of β calculated theoretically for the compounds.

When compared to urea as a reference substance, all the compounds examined have higher polarizability and first order hyperpolarizability values, indicating that they are likely to be good NLO substances. These data are comparable with that recently published for related systems. This suggests that they would be good NLO substances^44–47^.

### Molecular electrostatic potential surfaces (MEP)

The MEP determines whether a portion of the molecule is attracted or repulsive to a proton positioned at any location around the molecule^48^.

**Figure 4:**
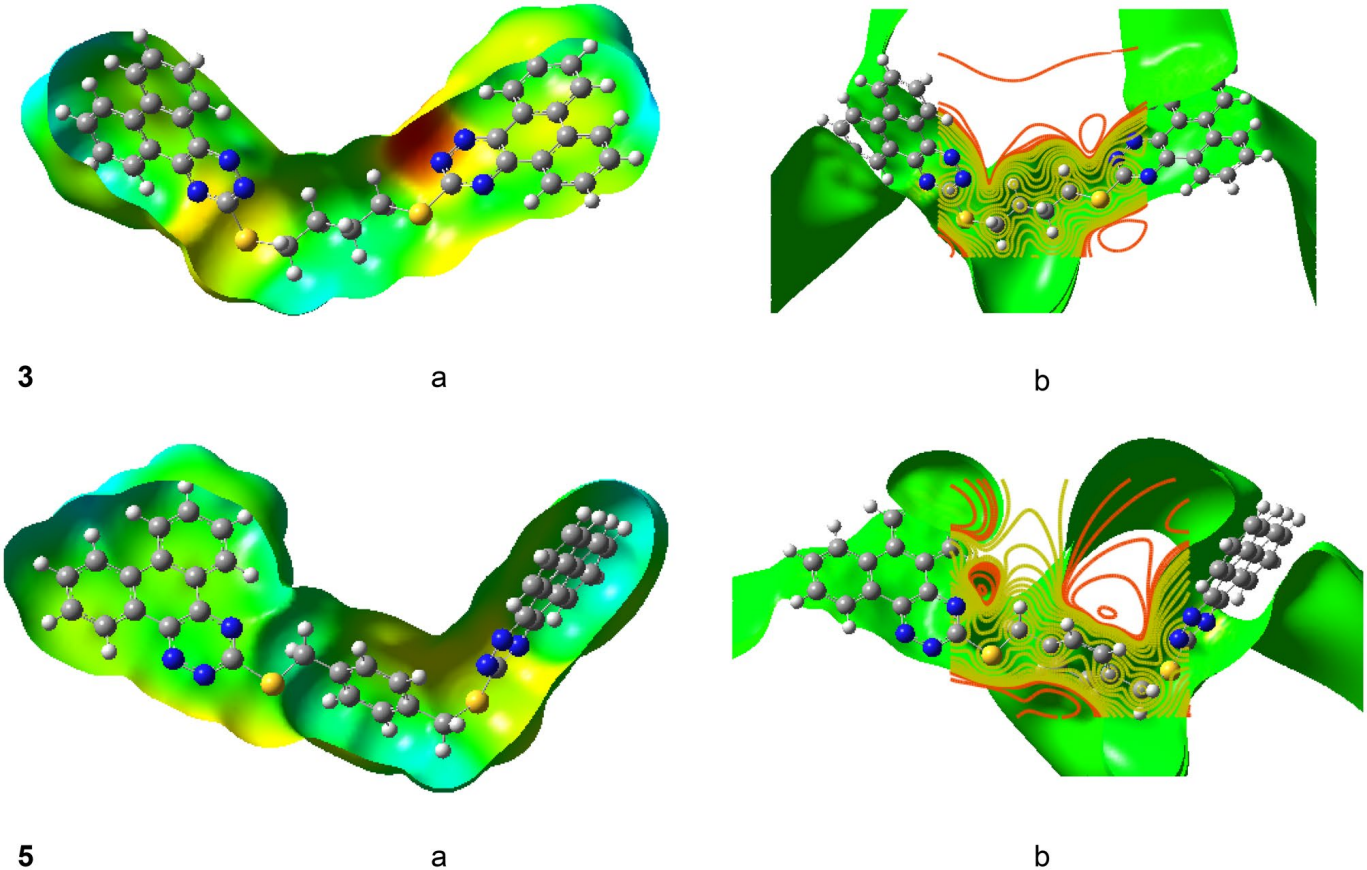
Molecular electrostatic potential (**a**) and electrostatic potential (**b**) surfaces of **3** and **5** using B3LYP/6-311G^∗∗^.

The DFT technique (B3LYP) and basis set (6-311G**) were used to optimize the geometry of the MEP surfaces, as shown in Fig. 4. Red represents an electron-rich, partially negative charge; blue represents an electron-deficient, partially positive charge; light blue represents a slightly electron-deficient zone; yellow represents a slightly electron-rich region, the green represents neutral (zero potential)^49^. A positive area (blue) in the corners of the MEP plot distinguishes the studied compounds **3** and **5**. The N atoms of the triazine moiety of **3** and **5** are responsible for the negative charge area.

The regions with the negative potential are over the electronegative atoms (N atoms), as indicated in MEP of the examined **3** and **5** compounds in Fig. 4, and regions of negative electrostatic potential are generally associated with the lone pair of electronegative atoms. The triazine moiety has the largest negative potential, whereas the hydrogen atoms have the highest positive potential. The potential of the carbon atoms appears to be zero. The triazine moiety of the two compounds has the most reactive sites, as indicated by MEP and electrostatic potential surfaces in Fig. 4.

### TD-DFT studies

TD-DFT calculations were brought out at the same level of theory (B3LYP/6-31G(d,p)) to clarify the origin of electronic spectra, using the polarized variable continuum solvation method, PCM, PCM-TD-DFT. In PCM the solute part remains inside the cavity, whereas the solvent part (ethanol) is denoted as a structureless material. The solvent is also illustrated in the PCM method by its dielectric constant and other macroscopic parameters. TD-DFT calculations of **6** and **8** were carried out on models representing their molecular structures. The theoretical spectrum of **3** is characterized by three bands at 436, 434, and 386 nm corresponding to HOMO-2 → LUMO + 1, HOMO-4 → LUMO, and HOMO 1 → LUMO/HOMO → LUMO + 1 in that order. The transition at 436 nm corresponds to a 70.13% contribution from the HOMO-2 → LUMO + 1 (n–π*) transition, whereas the second excitation band at 434 nm is due to a 69.22% contribution.

**Figure 5:**
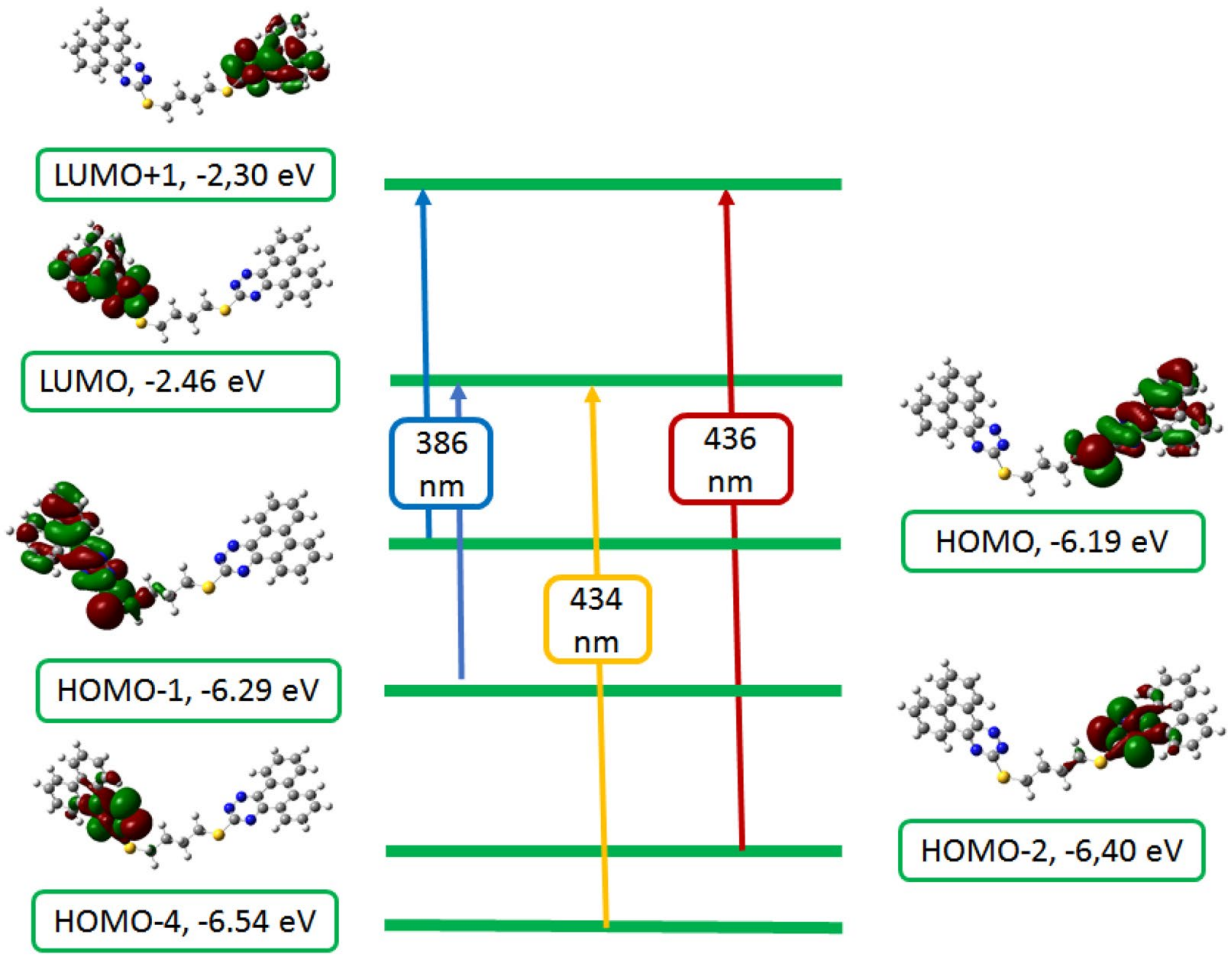
Frontier molecular orbitals involved in the electronic absorption transitions of compound **3** calculated at TD-B3LYP/6-31G(d,p) level of theory.

**Figure 6:**
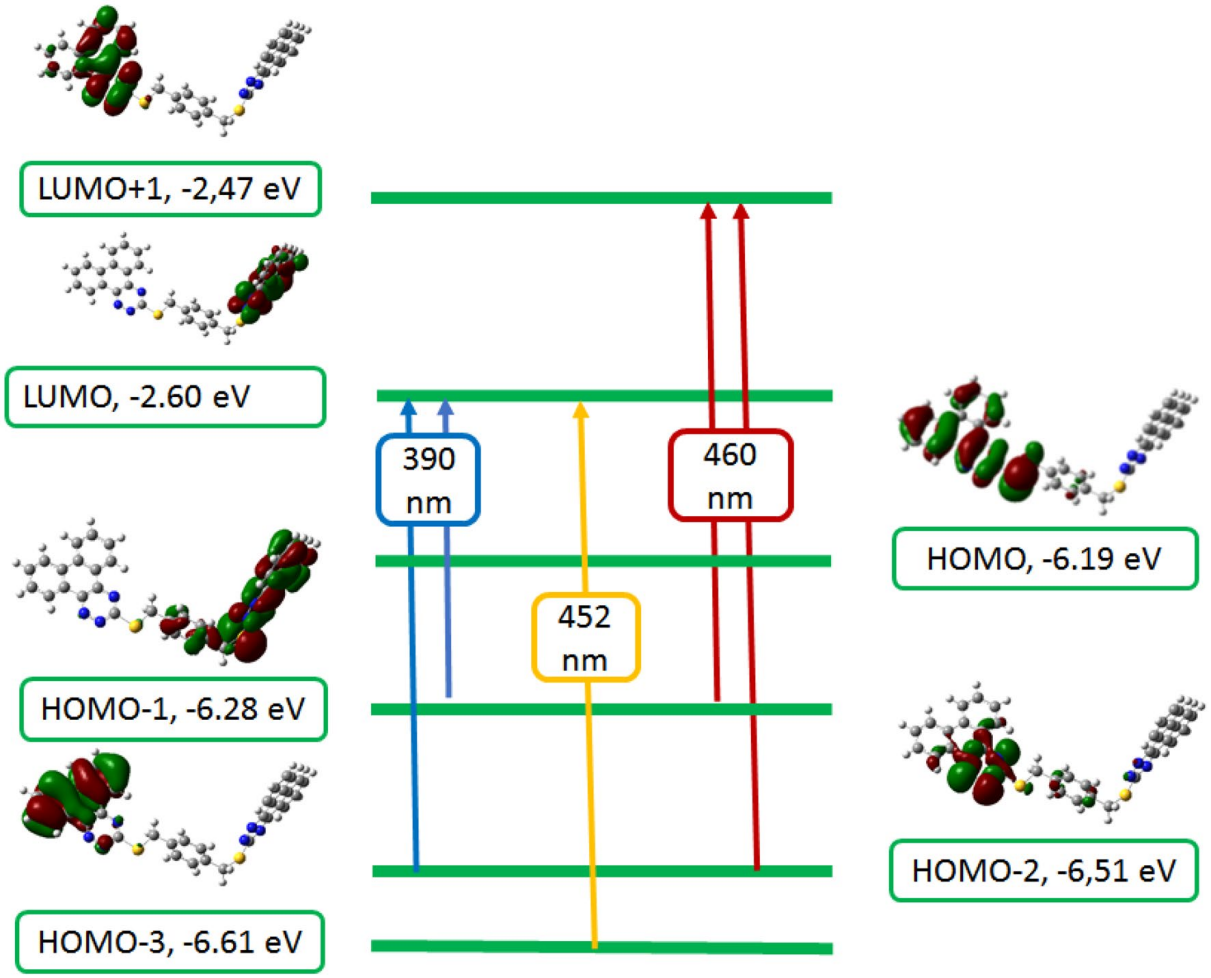
Frontier molecular orbitals involved in the electronic absorption transitions of compound **5** calculated at TD-B3LYP/6-31G(d,p) level of theory.

HOMO-4 → LUMO, (π–π*) transition, the third excitation band at 386 nm is corresponding to 25.25% contribution, HOMO- → LUMO (π–π*) transition. Hence, the vertical excitation energy states are S0 → S3, S0 → S4, S0 → S2, respectively, and are the only allowable transition states with effective oscillator strengths in ethanol. The explanations of FMO's orbitals and transfer of the electron density of compound **3**, which are involved in the electronic transitions are given in (Fig. 5). Three bands are observed in the TD-DFT spectrum (Fig. 6) of compound **5** at 460, 452, and 390 nm due to the following transitions: HOMO-1/HOMO-2 → LUMO + 1, HOMO-3 → LUMO, and HOMO-1/HOMO-2 → LUMO. The transition at 460 nm is matching to 48.95% contributive from HOMO-1/HOMO-2 → LUMO + 1 (n–π*) transition, while the second excitation band at 452 nm corresponds to 68.91% contribution, HOMO-3 → LUMO, (π–π*) transition, the third excitation band at 390 nm is related to 51.07% contribution, HOMO-1/HOMO-2 → LUMO, (π–π*) transition. The vertical excitation energy states are S0 → S3, S0 → S4, and S0 → S1, and respectively, are the only permitted transition states with great oscillator strengths in ethyl alcohol.

### Spectroscopic comparison of compounds (3 and 5) as well as compounds (6 and 7)^14^ in ethanol

**Figure 7:**
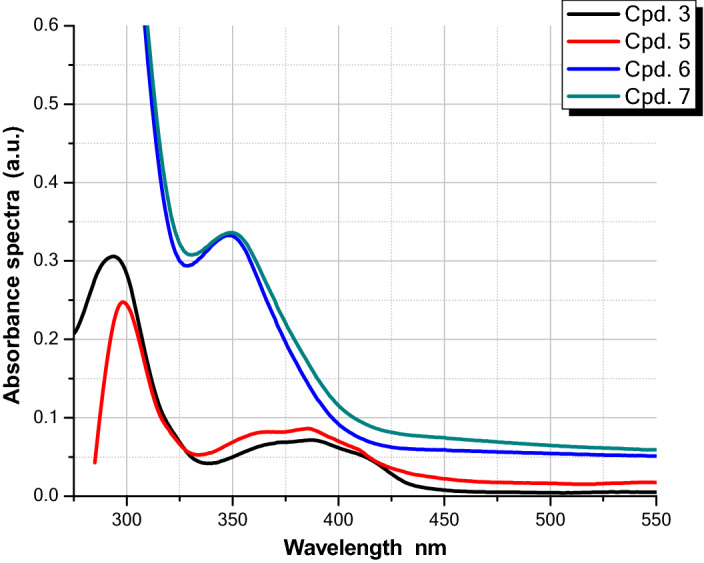
Absorption spectra of 2 × 10^–5^ M of different triazine chromophores in ethanol.

The redshift of the absorption profile of compounds **3** and **5** relative to **6** and **7** may be due to the more conjugated structures of fused triazine chromophores than those of phenyl-substituted triazines, as seen in absorption spectra of various triazine-based chromophores (Fig. 7).

**Figure 8:**
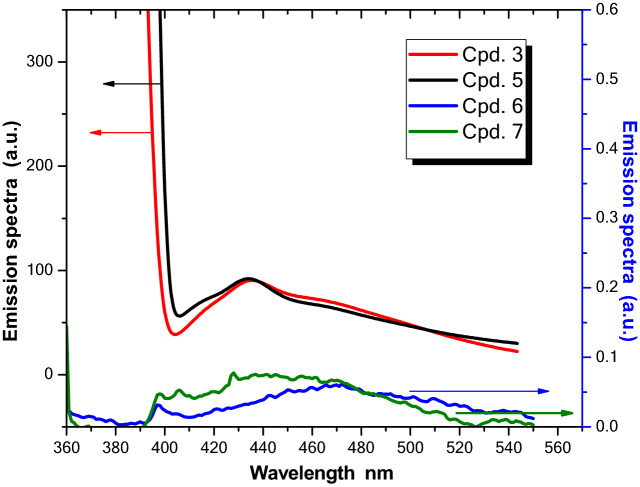
Emission spectra of 2 × 10^–5^ M of different triazine chromophores in ethanol.

Furthermore, due to the significant non-radiative radiation of the diphenyl substituted triazine chromophore, the emission spectra of compounds **3** and **5** (Fig. 8) displayed higher fluorescence (more than 10^3^ times) compared to non-fused triazines **6** and **7.** This would enhance the value of compounds **3** and **5** in technological applications.

### Solvent’s effect on absorption and emission spectra of fused triazines 3 and 5

**Figure 9:**
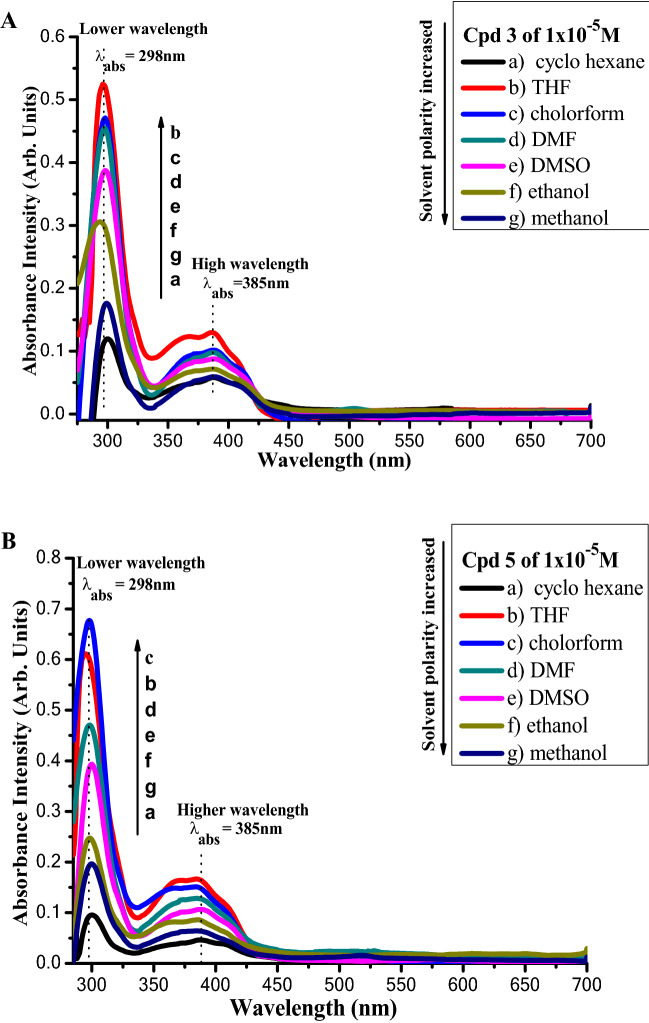
(**A**,**B**).Absorption of compounds** 3** and **5** of concentration 1 × 10^−5^ M in different solvents.

**Figure 10:**
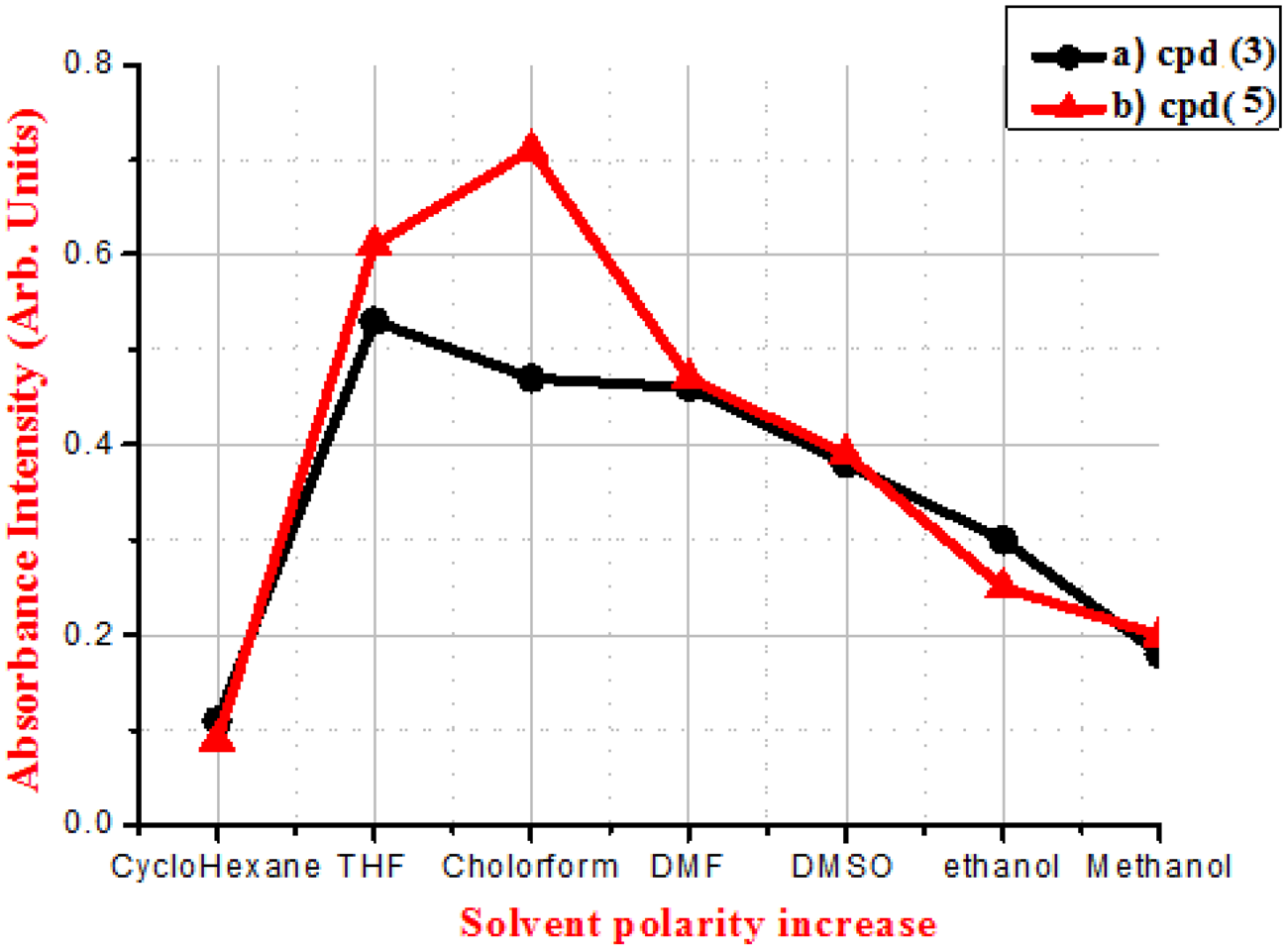
Absorption intensities of compounds **3** and **5** of concentration 1 × 10^−5^ M in different solvent polarities.

**Figure 11:**
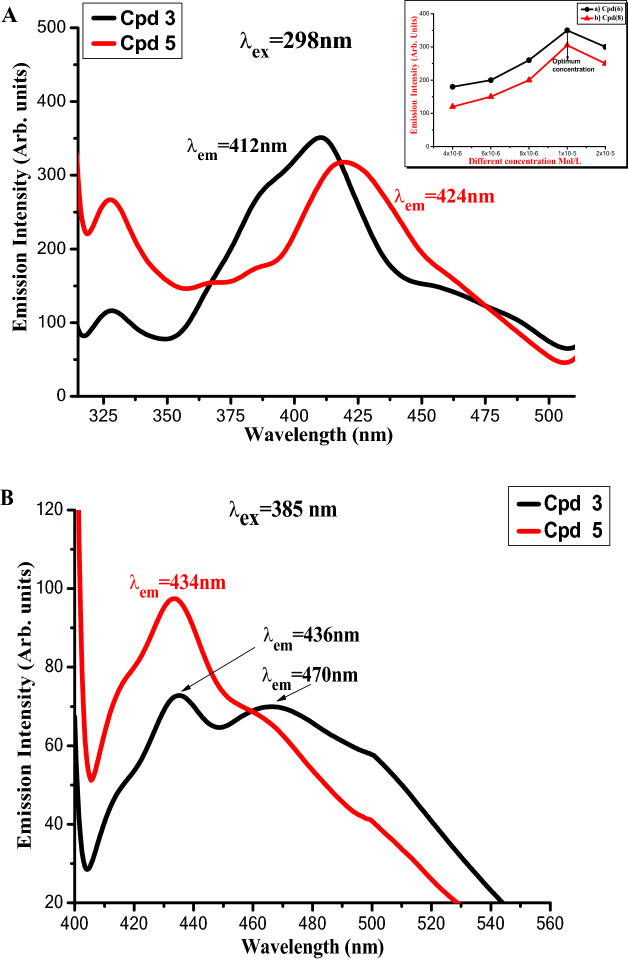
Emission of compounds **3** and **5** of concentration 1 × 10^−5^ M in DMF A) at lower excitation λ_ex_ = 298 nm (the inset figure is the emission intensities of different concentrations in DMF). B) At higher excitation λ_ex_ = 385 nm.

Figures 9, 10 and 11 illustrate the absorption and fluorescence spectra of fluorophores **3** and **5** in various solvents with varied solvent parameters such as dielectric constant (ш), refractive index (n), and H-bond power. Solvents a utilized range in polarity from non-polar to aprotic polar to protic polar.

Figure 9A,B shows that the absorption spectra of compounds **3** and **5** are nearly identical, with two maximum absorption peaks at 296, 385 nm, and 298, 385 nm in compounds **3** and **5**, respectively.

The absorption intensity of compound 5 is somewhat higher than that of compound **3**, which might be due to a variation in the spacer, causing compound **5** to rotate more slowly.

Figure 10 shows the absorption intensities of compounds **3** and **5** in various solvents. The absorption intensities of both dyes are lowest in cyclohexane, a non-polar solvent.

In polar media, on the other hand, when the polarities of polar solvents rise, the absorption intensities drop, which is consistent with previously held beliefs.

Figure 11A,B shows the emission spectra of compounds **3** and **5** at lower and higher excitation of concentration 1 × 10^−5^ M, while the inset figure in Fig. 11A shows the emission intensity of compounds **3** and **5** at various concentrations, indicating that 1 × 10^−5^ M is the optimal concentration.

Compound **5** showed a higher fluorescence intensity as well as a better fluorescence efficiency (in terms of area under emission spectra) than compound **3**, which might be due to its limited rotation, which reduces non-radiative relaxation processes that compete with fluorescence. In addition, the emission profile of compound **3** exhibits a wider band with two maxima peaks at wavelengths 436 nm and 470 nm, which might be attributable to the presence of an alkyl spacer leading to free geometrical orientation, as seen in Fig. 11B.

**Figure 12:**
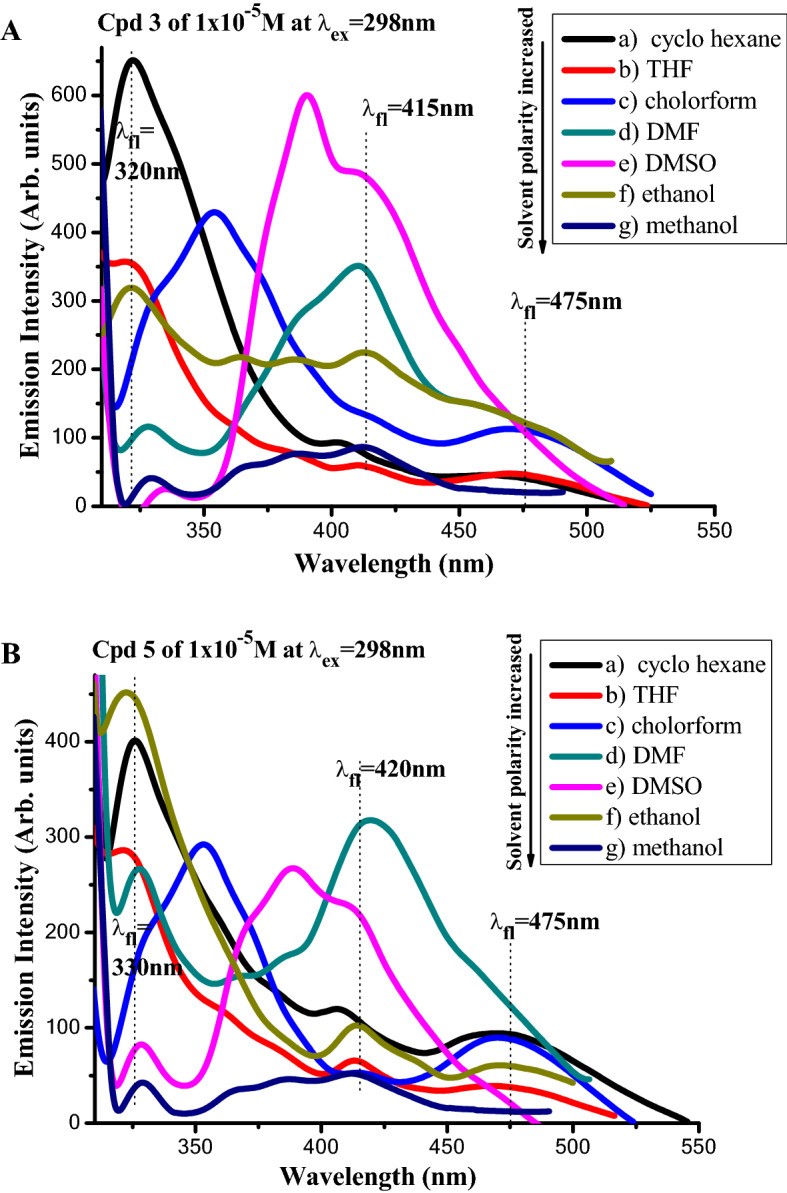
(**A**,**B**) Emission spectra of 1 × 10^−5^ M of compounds (a) **3**, (b)** 5** exciting wavelength (298 nm) in different solvents.

**Figure 13:**
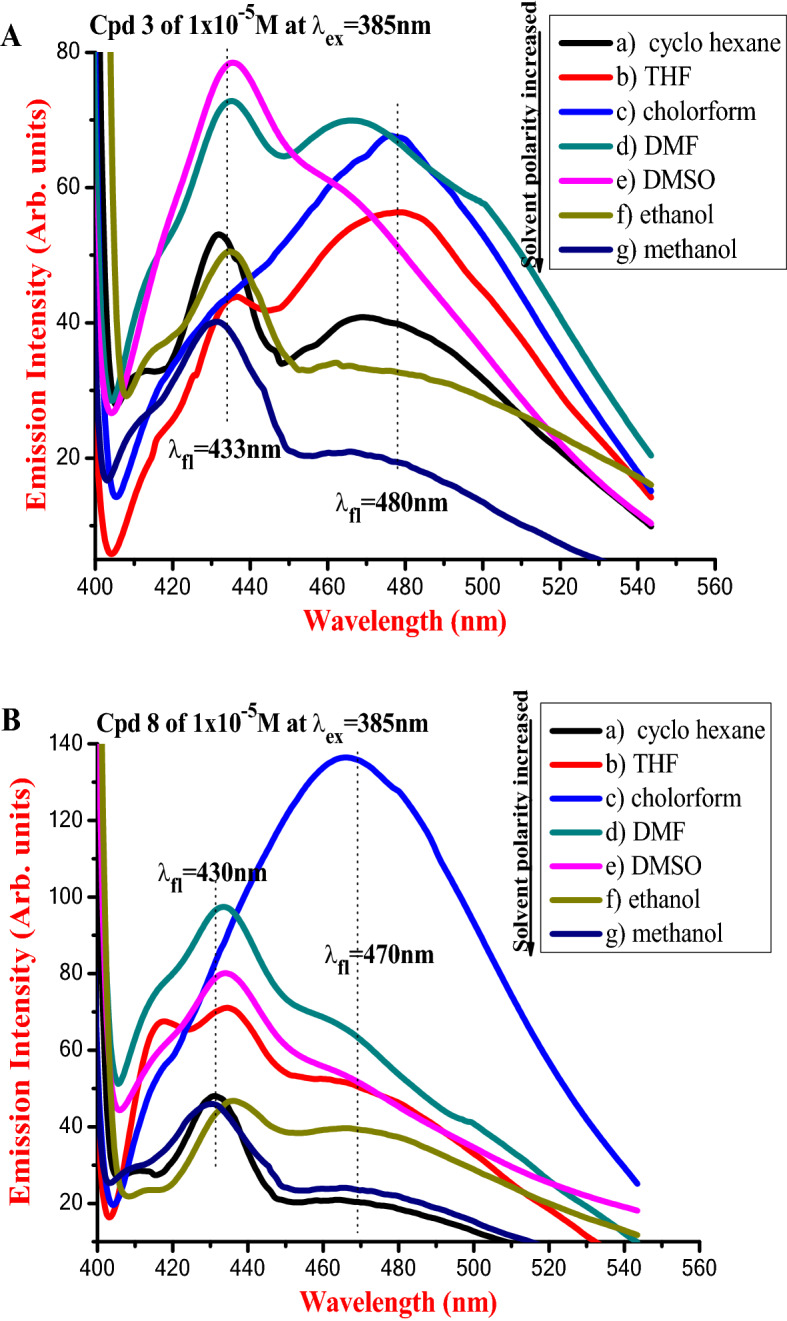
(**A**,**B**) Emission spectra of [1 × 10^−5^ M] of compounds (a) **3**, (b) **5** of exciting wavelength (385 nm) in different solvents.

Measuring the emission spectra of compounds **3** and **5** in different solvents, as shown in Fig. 12A,B by lower excitation wavelength (298 nm) and Fig. 13A,B by higher excitation wavelength (385 nm), revealed that the different solvents had a significant impact on the new dyes' spectral properties. In each solvent type, the emission profiles of compound **3** (shown in Fig. 12A) revealed varied peak intensities.

The spectra contain two peaks at 320 nm and 403 nm in a non-polar solvent (i.e., cyclohexane). The redshift of emission spectra was noticed when the polarity index of the aprotic polar solvent rose. Finally, protic polar solvents with strong polarity (such as ethanol and methanol) exhibited a wide emission profile with a variety of hump highs. Hydrogen bond formation may be to blame for the later profile behavior. Excitation of compound **3** with a long wavelength (385 nm) (Fig. 13A) resulted in a wide emission profile with two major peaks at about 431–434 nm and 450–480 nm, with varying intensities depending on the composition of the solvent. Figures 12B and 13B show the emission patterns of compound **5** based on the same contemporaneous behavior as compound **3**. When stimulated by 385 nm wavelength, the emission spectra of chemical **5** dissolved in DMF as an aprotic polar solvent showed an exception. In comparison to other profiles, this profile exhibits the broadest and strongest redshift at 470 nm. In other solvents, the highest emission peak was about 430 nm. Single and double benzene rings are associated with a strong peak at an emission wavelength less than 350 nm, while polycyclic aromatic compounds are associated with an emission wavelength higher than 380 nm^50,51^.

Overall, when the solvent polarity rose, the emission peaks shifted to the long wavelength (redshift) of the spectra, with widening in the fluorescence band, implying that the singlet excited state is more polar than the ground state.

As a result, the emission spectra are more influenced by the polarity of the solvent than the absorption spectra, indicating that a significant charge transfer was occurring in the excited state. Finally, the novel dyes' photophysical characteristics were reported, summarized in Table 4 (A and B), and demonstrated their potential use in advanced optical applications.

**Table 4:** (A, B) Photophysical parameters of compounds **3** and **5**, respectively; (ε) molecular extinction coefficient; σ_a_ and σ_e_: absorption and emission cross-sections; (Λ) the attenuation length, (τ_call_) calculated fluorescence lifetime, μ_12_(D) the transition dipole moment, (E_f_) energy yield of fluorescence, (K_r_) the radiative decay rate, (K_isc_) the intersystem crossing rate, (f) oscillator strength, φ_f_ fluorescence quantum yield.

Solvent	ε L M^1^ Cm^−1^ (10^4^)	σ_a_ (10^–16^) Cm^2^	σ_e_ (10^–19^) Cm^2^	Λ (cm)	τ_0_ (ns)	τ_f_ (ns)	μ_12_ (D)	E_f_	K_r_ (10^9^) s^−1^	K_isc_ (10^9^) s^−1^	F	φ_f_
(**A) Cpd. 3**												
Cyclohex-ane	1.10	0.42	0.24	0.39	0.32	0.115	3.91	0.39	3.12	5.82	0.12	0.33
THF	5.30	2.04	0.62	0.08	0.25	0.100	4.76	0.46	4.00	6.00	0.48	0.40
Chloroform	4.70	1.81	0.41	0.09	0.23	0.096	4.02	0.50	4.34	6.04	0.39	0.42
DMF	4.60	1.77	0.53	0.09	0.21	0.095	5.45	0.49	4.75	5.78	0.44	0.45
DMSO	3.80	1.46	0.65	0.11	0.20	0.098	5.28	0.58	5.00	5.20	0.42	0.49
Ethanol	3.10	1.19	0.26	0.14	0.28	0.101	4.26	0.41	3.57	6.33	0.35	0.36
Methanol	1.80	0.69	0.23	0.24	0.3	0.105	4.91	0.27	3.33	6.19	0.19	0.35
**(B) Cpd. 5**												
Cyclohexane	1.00	0.38	0.04	0.43	0.65	0.20	4.38	0.26	1.53	3.4	0.11	0.32
THF	6.1	2.35	0.23	0.07	0.78	0.29	5.35	0.41	1.28	2.13	0.70	0.38
Chloroform	7.1	2.73	0.55	0.06	0.62	0.32	6.06	0.47	1.61	1.50	0.65	0.52
DMF	4.7	1.81	0.48	0.09	0.53	0.25	5.75	0.49	1.88	2.08	0.26	0.48
DMSO	3.9	1.50	0.41	0.11	0.47	0.21	6.82	0.54	2.12	2.57	0.18	0.46
Ethanol	2.5	0.96	0.05	0.17	0.61	0.21	4.99	0.38	1.63	3.09	0.51	0.35
Methanol	2	0.77	0.09	0.21	1.02	0.33	4.74	0.35	0.98	2.03	0.15	0.33

## Conclusion

The production of various new fluorescent derivative dyes was addressed in this study. At the B3LYP/6-311**G(d,p) basis set, the optimal molecular structures of novel phenanthrotriazines were obtained. Recent studies have shown that phenanthrene, 1,2,4-triazines, and their fused derivatives are viable candidates for nonlinear applications. This behavior was validated by calculating the total molecule dipole moment, linear polarizability, and hyperpolarizability. These nonlinearities are caused by highly delocalized π-electron in these systems. To extend the conjugation, our research involved the synthesis of novel fused systems, bis(phenanthro[9,10-*e*][1,2,4]triazines), connected by flexible aliphatic or rigid aromatic moieties. The compounds may be readily polarized and have significant NLO characteristics, as seen by the tiny HOMO–LUMO energy gap. The compounds' polarizability and hyperpolarizabilities characteristics indicate that they are capable of being used as NLO materials. Using urea as a reference substance, both examined triazine derivatives have higher polarizability and first order hyperpolarizability values (Compound **5** is 24 times higher than urea, whereas compound **3** is 15 times higher) indicating that they are likely to be good NLO substances. The fluorescence quantum yield of compound **3** with a butylene spacer was found to be lower than compound **5** with an aromatic spacer which may be attributed to the facilitation of a butylene spacer for free rotation. The influence of polarity and the intermolecular charge transfer processes are reflected in the differences in quantum yields and fluorescence lifetimes in various solvents (ICT).

The emission spectra of fused triazine-based compounds **3** and **5** displayed higher fluorescence (more than 10^3^ times) compared to non-fused triazines **6** and **7**. This would increase the utility of the new fused-triazine compounds in optical technology applications.

These findings stimulate future theoretical and experimental studies, such as enlarging the conjugated network by adding some electron-donating and electron-withdrawing groups to the phenanthrotriazine system, which would correspond to the so-called push–pull effect. This would result in a bathochromic shift of their UV/vis absorption and emission bands, as well as increased molar extinction coefficients, and therefore improve their nonlinear characteristics (Supplementary Information).

## Supplementary Information

Supplementary Information.

## Data Availability

The datasets used and/or analyzed during the current study are available from the corresponding author upon reasonable request.
