# Supplementary material for: Experimental and theoretical studies of linear and non-linear optical properties of novel fused-triazine derivatives for advanced technological applications
Source: Sci Rep. 2022 Nov 19;12:19937. doi: 10.1038/s41598-022-22311-z (PMC9675782; doi:10.1038/s41598-022-22311-z)

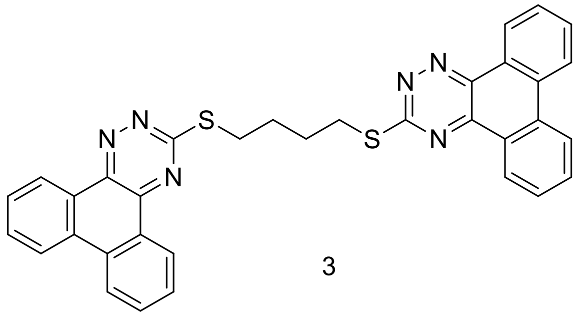

3

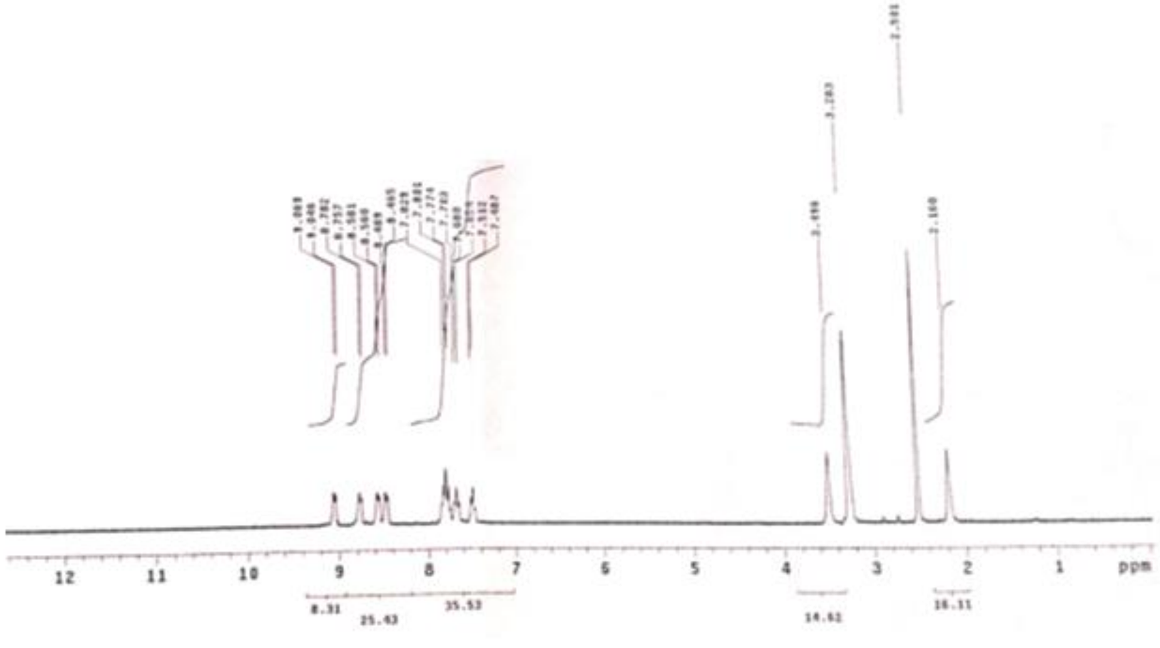

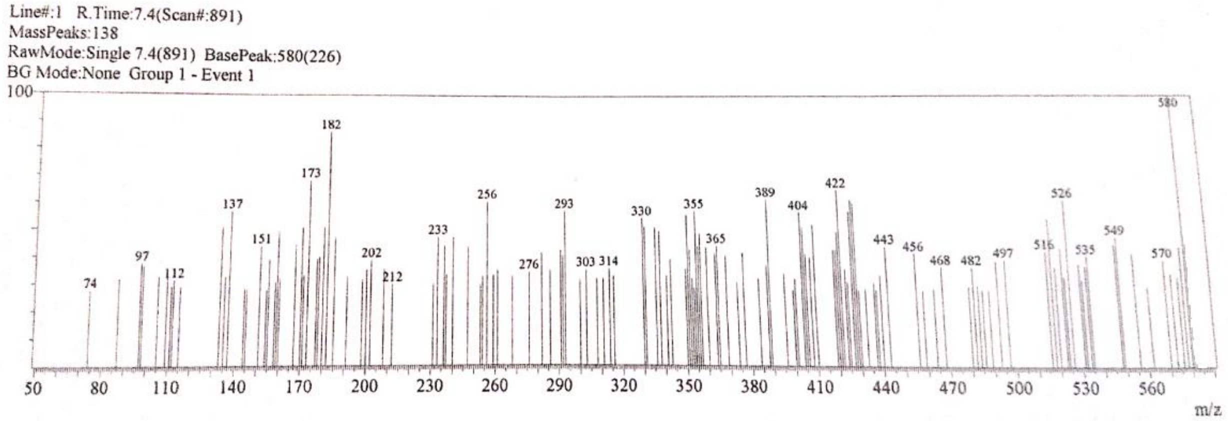

Line#:1 R.Time:7.4(Scan#:891)
MassPeaks:138
RawMode:Single 7.4(891) BasePeak:580(226)
BG Mode:None Group 1 - Event 1
100
74
97
112
137
151
173
182
202
212
233
256
276
293
303 314
330
355
365
389
404
422
443
456
468
482
497
516
526
535
549
570
580
50 80 110 140 170 200 230 260 290 320 350 380 410 440 470 500 530 560
m/z

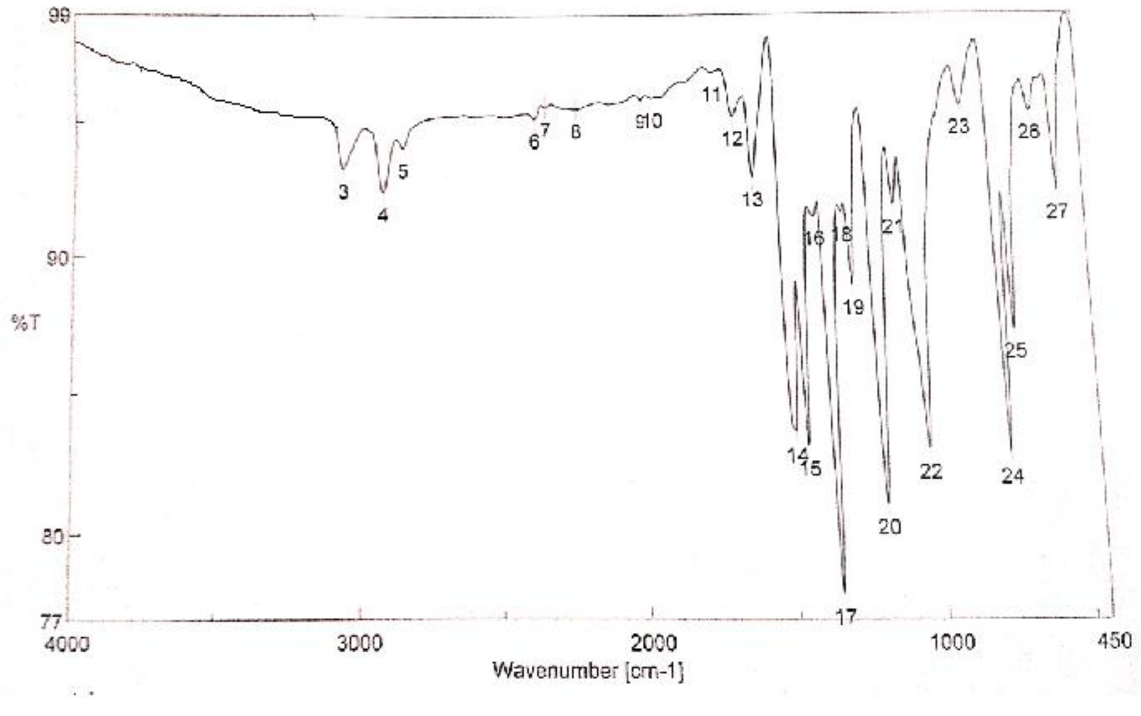

99
90
80
77
%T
4000 3000 2000 1000 450
Wavenumber [cm-1]

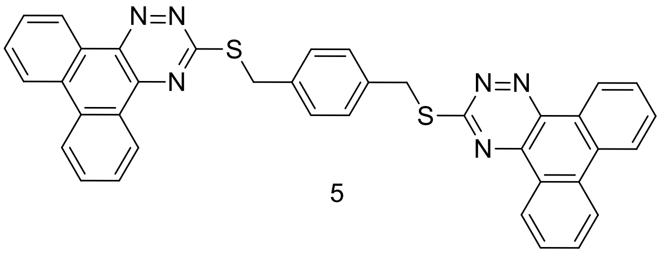

5

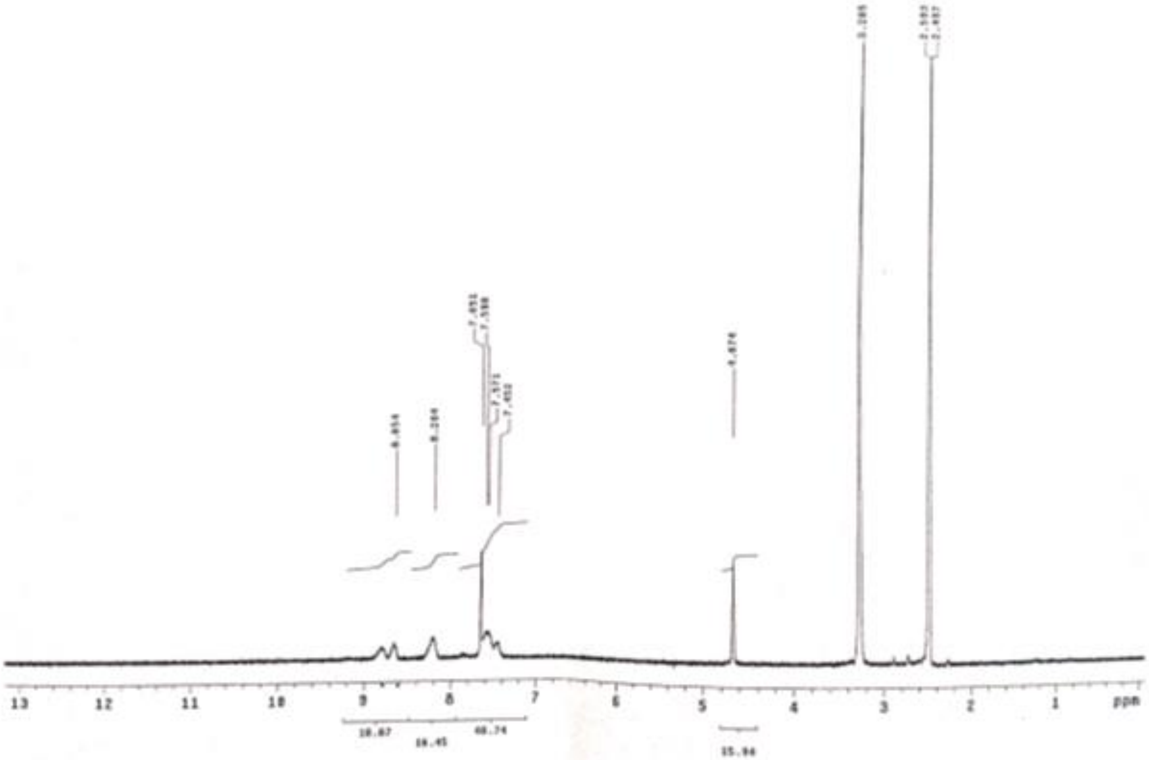

Line#:1 R.Time:2.0(Scan#:241)
MassPeaks:161
RawMode:Single 2.0(241) BasePeak:228(158)
BG Mode:None Group 1 - Event 1

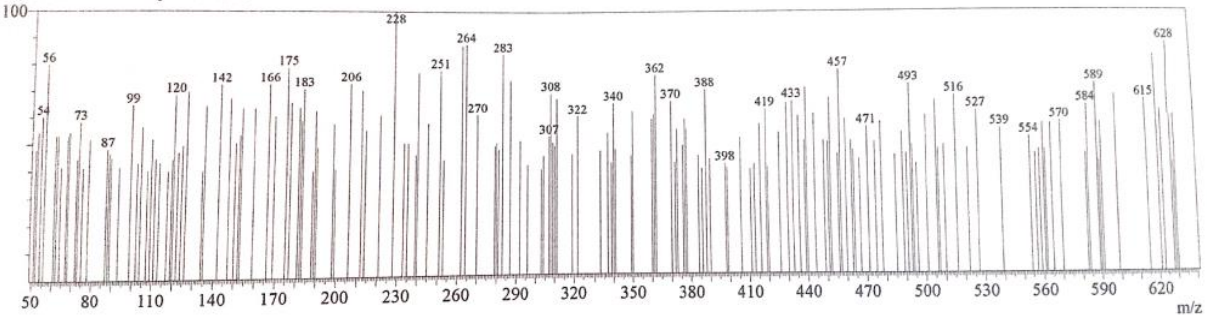

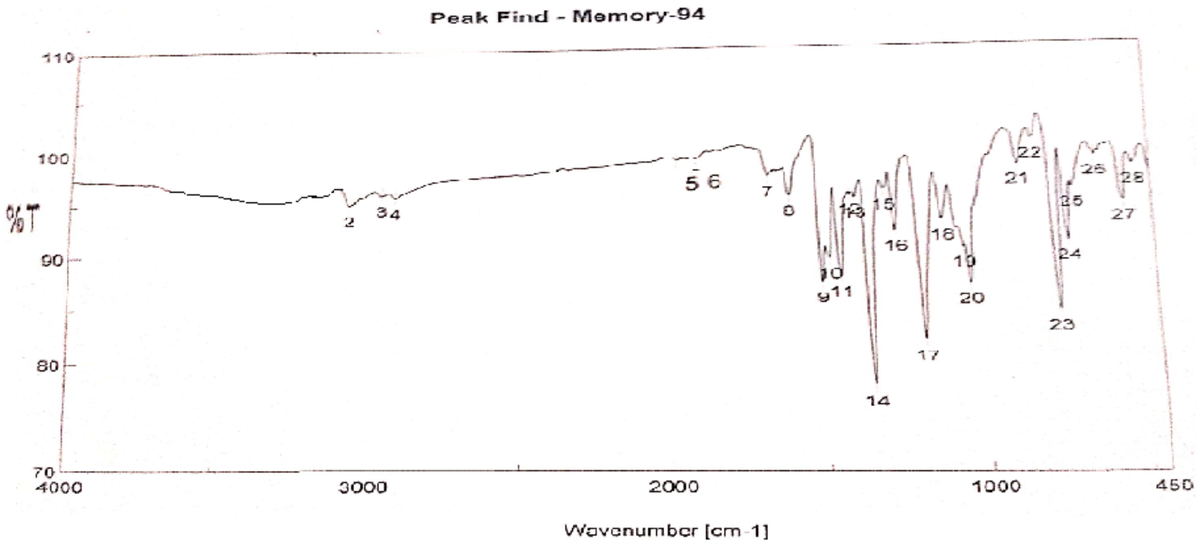

Supplement: Supplementary file 1 — Supplementary Information. [file 41598_2022_22311_MOESM1_ESM.pdf]
